# Pre-pubertal accelerometer-assessed physical activity and timing of puberty in British boys and girls: the Millennium Cohort Study

**DOI:** 10.1093/ije/dyad063

**Published:** 2023-05-19

**Authors:** Tuck Seng Cheng, Soren Brage, Esther M F van Sluijs, Ken K Ong

**Affiliations:** MRC Epidemiology Unit, Institute of Metabolic Science, University of Cambridge School of Clinical Medicine, Cambridge, UK; MRC Epidemiology Unit, Institute of Metabolic Science, University of Cambridge School of Clinical Medicine, Cambridge, UK; MRC Epidemiology Unit, Institute of Metabolic Science, University of Cambridge School of Clinical Medicine, Cambridge, UK; MRC Epidemiology Unit, Institute of Metabolic Science, University of Cambridge School of Clinical Medicine, Cambridge, UK; Department of Paediatrics, University of Cambridge, Cambridge, UK

**Keywords:** Accelerometer, physical activity, puberty timing, substitution analysis, compositional analysis

## Abstract

**Background:**

Early puberty timing is associated with adverse health outcomes. We aimed to examine prospective associations between objectively measured physical activity and puberty timing in boys and girls.

**Methods:**

In the UK Millennium Cohort Study, physical activity volume and intensities at 7 years were measured using accelerometers. Status of several pubertal traits and age at menarche were reported at 11, 14 and 17 years. Age at menarche in girls was categorized into tertiles. Other puberty traits were categorized into earlier or later than the median ages calculated from probit models, separately in boys and girls. Multivariable regression models, with adjustment for maternal and child characteristics including body mass index (BMI) at age 7 years as potential confounders, were performed to test the associations of total daily activity counts and fractions of activity counts across intensities (in compositional models) with puberty timing, separately in boys (*n = *2531) and girls (*n = *3079).

**Results:**

Higher total daily activity counts were associated with lower risks for earlier (vs later) growth spurt, body hair growth, skin changes and menarche in girls, and more weakly with lower risks for earlier skin changes and voice breaking in boys (odds ratios = 0.80–0.87 per 100 000 counts/day). These associations persisted on additional adjustment for BMI at 11 years as a potential mediator. No association with puberty timing was seen for any physical activity intensity (light, moderate or vigorous).

**Conclusions:**

More physical activity regardless of intensity may contribute to the avoidance of earlier puberty timing, independently of BMI, particularly in girls.

Key Messages

- Higher total volume of physical activity was associated with ∼20% lower risks for earlier (than the cohort average) timing of several pubertal traits, particularly in girls.
- The associations between total volume of physical activity and puberty timing were independent of pre-pubertal and pubertal body mass index.
- Separately from total physical activity, light-, moderate- and vigorous-intensity activities were not associated with puberty timing.

## Introduction

Puberty is the process of physical and physiological changes from childhood to adulthood to achieve reproductive maturity. It begins with the appearance of breast and genital development and progresses to first menstruation (i.e. menarche) and voice breaking in girls and boys, respectively.^1–4^ These physical changes of puberty occur at a wide range of ages from 8 to 14 years^4^^,^^5^ and the whole process typically takes 2–3 years. Puberty now starts at an earlier age than previously in most populations worldwide.^6^^,^^7^ The timing of puberty is of particular interest because earlier puberty is associated with higher risks for adverse mental and metabolic health outcomes^8–12^ and breast cancer.^10^^,^^13^ A systematic review estimated that ∼13% of type 2 diabetes was attributable to early menarche timing among White British women.^9^ The underlying mechanisms may partly involve obesity that precedes early puberty,^14^^,^^15^ suggesting that puberty timing may be modifiable by pre-pubertal adiposity-related behavioural factors. As well as dietary intake,^16–18^ pre-pubertal physical activity may influence later body composition^19^ and puberty timing.^20^

A recent review found only a few studies that have examined the prospective association between physical activity and puberty timing.^20^ Furthermore, those reports were limited to menarche status in girls.^20^ In the only identified randomized–controlled trial, a successful school-based obesity-prevention intervention delayed the attainment of menarche in girls.^21^ However, that intervention changed both diet and physical activity components, and so it was difficult to disentangle the specific effect of physical activity on age at menarche.^20^^,^^21^ Moreover, one prospective cohort study found that higher weight-adjusted energy expenditure and physical activity duration were associated with lower risk for earlier menarche^22^ but these associations were not observed in other studies.^23–26^ Notably, those previous studies were limited to self-reported overall physical activity, which may be subject to measurement errors. It is also increasingly recognized that not just the total volume of physical activity but also its component intensities may play a role in cardiometabolic risk factors among children.^27^ To our knowledge, no study has yet analysed device-measured physical activity nor its intensities in relation to puberty timing. Further, physical activity was previously assessed among pre-menarcheal girls at ages when puberty had likely already started, and menarche status was reported after short follow-up durations of 1–3 years,^22–26^ which raises the possibility of reverse causality (i.e. reductions in physical activity following the onset of puberty). A meta-analysis of 13 case–control studies reported that female athletes had 1.13 years later age at menarche than non-athletes;^20^ however, this finding is likely to be confounded by other differences between those groups, such as diet and weight regulation.^20^ Therefore, it is unclear whether total physical activity or any particular intensity influences puberty timing directly and whether any such effects are mediated by body mass index (BMI). Identifying the role of physical activity including its intensity in pubertal development is important in better informing future intervention and/or preventive strategies for early puberty and/or related diseases especially among high-risk children.

In the present study, we investigated the prospective associations between physical activity and timings of several puberty traits in boys and girlsin the UK Millennium Cohort Study (MCS) using simultaneous modelling of volume and intensity in order to distinguish their independent contributions. This large prospective cohort used accelerometers to measure physical activity at age 7 years (prior to the age of physiological puberty onset) and repeated self-assessments of pubertal development. We adjusted models for baseline BMI and further explored whether any observed association might be mediated by BMI at age 11 years. We hypothesized that more physical activity would be associated with later puberty timing, possibly dependent on or independent of change in BMI.

## Methods

### Study participants

The MCS is an on-going nationally representative, longitudinal cohort study that enrolled more than 19 000 children born between September 2000 and January 2002 in the UK.^28^^,^^29^ Eligible children were first identified using government child benefit records, a benefit with almost universal coverage, and recruited at age 9 months. A small number of eligible children who were missed at initial recruitment were further included at subsequent visits. Data collection on diverse topics including behaviour and physical growth have been conducted at age 9 months (MCS1) (*n = *18 818) and 3 (MCS2) (*n = *15 808), 5 (MCS3) (*n = *15 460), 7 (MCS4) (*n = *14 043), 11 (MCS5) (*n = *13 469), 14 (MCS6) (*n = *11 872) and 17 (MCS7) years (*n = *10 757). Informed consent was obtained from parents as well as from the children themselves as they aged. Further details including available data can be found at https://cls.ucl.ac.uk/cls-studies/millennium-cohort-study/.

The ethical approval for this cohort study was obtained from the South West Multi-Centre Research Ethics Committee (MCS1), the London Multi-Centre Research Ethics Committee (MCS2 and MCS3), the Northern and the Yorkshire Research Ethics Committee (MCS4), the Yorkshire and Humber Research Ethics Committee (MCS5), the National Research Ethics Service Research Ethics Committee London—Central (MCS6) and the National Research Ethics Service Research Ethics Committee North East—York (MCS7).

### Physical activity measures

Physical activity (15-s sampling epochs—the shortest possible epoch given the number of wearing days) was measured at age 7 years (mean ± SD: 7.23 ± 0.25 years) (MCS4) using an accelerometer (Actigraph GT1M, Pensacola, Florida) worn on the right hip. The accelerometer is a small, lightweight and non-waterproof device, and has been extensively validated in children against observational techniques,^30^ heart rate monitoring,^31^ indirect and room calorimetry^32^^,^^33^ and doubly labelled water techniques.^34^ All children surveyed at age 7 years were invited and participating children with written consent from their parents (*n = *13 219, 94.1%) were posted an accelerometer and instructed to wear it (from the morning after they received it) on an elastic belt around their waist during waking time (except during bathing and other aquatic activities) for up to 7 consecutive days (without restriction on any weekday or weekend). Parents were also asked to complete a physical activity timesheet during the monitoring period to record additional information including times spent on swimming and cycling. After the monitoring period, families returned their accelerometer and timesheet in a prepaid envelope (*n = *10 034).

Data processing from all returned, worn accelerometers was performed, as detailed elsewhere.^19^^,^^35^ In brief, accelerometry data (*n = *8939; of which 8699 were singletons) were downloaded using the ActiLife Lifestyle Monitoring System software version 3.2.1. Non-wear time was identified as any period of consecutive zero-counts for ≥20 min and excluded from the summation of activity.^36^ The accelerometry data were summarized as the overall movement volume, expressed as daily total counts, and time spent in sedentary [<100 counts per minute (CPM)], light (100–2240 CPM), moderate (2241–3840 CPM) and vigorous (>3840 CPM) intensities based on previously described thresholds,^37^ whereas extreme high values (≥11 715 CPM) were considered to be artefactual and were removed.^38^ Reliable data were defined as children with a monitor wear time period of ≥2 days, each day containing ≥10 h of valid wear data (*n = *6675; of which 6497 were singletons),^39^ whereas others had <2 days or <10 h per day of data (25.3%, *n = *2264 including 2202 singletons).

In order to analyse physical activity intensity beyond the overall volume, we derived fractions of total daily counts accumulated in each of the four intensity categories (Supplementary Figure S1, available as Supplementary data at *IJE* online). Using daily summary data (37 341 person-days of data in 6675 children), we first determined the average movement intensity (in CPM) across each intensity level from a single linear regression with interval constraints. The model included the empirical total daily counts as the dependent variable, times spent across four physical activity intensities as the independent variables and no constant value:


$$\text{empirical}\;\text{total}\;\text{daily}\;\text{counts}={\;\text{Time}}_{\text{sedentary}}+{\;\text{Time}}_{\text{light}}+{\;\text{Time}}_{\text{moderate}}+{\;\text{Time}}_{\text{vigorous}}$$

The resulting regression coefficients (i.e. average intensities) were pre-defined to be higher as the intensity level increased. We found that the average intensities for sedentary, light, moderate and vigorous activities were 0, 645, 3481 and 5754 CPM, respectively, which were within the above designated ranges.^37^ We further validated these average intensity estimates by comparing the estimated total counts (calculated by the summation of the products between time spent and derived average intensity) with the empirical total counts at the participant level. Estimated total counts were 0.19% higher than empirical total counts (mean difference ± standard deviation: 854 ± 18 200; paired *t*-test: *P *<* *0.001) but these values were highly correlated (Pearson’s correlation coefficient = 0.99). In each individual, and for each valid day, the fraction of total daily counts contributed by each intensity (in %) was calculated by dividing the estimated counts for each intensity by the estimated total daily counts. Next, in each individual, averages across all valid measure days were calculated for empirical total daily counts and fractions of total daily counts contributed by each intensity. Finally, these values were internally standardized by adjusting for age at measurement, total wear time, number of valid measure days, proportion of weekdays measured, season during measurement (coded as two orthogonal sine functions; ‘Winter’ peaking at 1 on 1 January and reaching a minimum of − 1 on 1 July, and ‘Spring’ peaking at 1 on 1 April and reaching a minimum of − 1 on 1 October).^40^

### Timings of puberty traits

Pubertal status in growth spurt, body hair and skin changes for boys and girls, voice breaking and facial hair only for boys, and breast development only for girls were reported by parents at 11 years (MCS5) and by children themselves at 14 years (MCS6) using a self-administered rating scale for pubertal development.^41^ The response options were: ‘has not yet begun’, ‘has barely started’, ‘has definitely started’ and ‘seems completed’. Menarche in girls was the only pubertal trait for which timing was directly reported: ‘How old was your child when she started to menstruate?’ and ‘How old were you when you had your first period?’ by parents at 11 years, and by girls themselves at 14 and 17 years (MCS4-MCS7), respectively.

To reduce inconsistencies between time points, we reclassified puberty status into binary categories at each time point: (i) pre-puberty—‘has not yet begun’ or ‘has barely started’ and (ii) puberty—‘has definitely started’ or ‘seems completed’. Among those with responses at both visits, the proportions of those with inconsistent reporting (i.e. puberty at one visit but pre-puberty at the next) were 0.4% for voice breaking, 0.6% for facial hair, 1.5% for body hair, 2.8% for skin changes, 5.5% for breast development and 12.3% for growth spurt, and these were removed from analyses of that particular pubertal trait (only three were removed for all traits). Because ages at pubertal assessment at each time point ranged widely from 10.2 to 12.3 years (MCS5; median = 11.2 years) and 13.1 to 15.3 years (MCS6; median = 14.3 years), likely after puberty had already started, individual puberty timing could not be confidently and directly derived from age at questionnaire completion when puberty in any trait is reported. Furthermore, associations between age at questionnaire completion with maternal education and family income (not shown) meant that use of time-to-event models may introduce additional bias. Therefore, we first estimated the median age for the report of each physical puberty trait across the whole sample using probit modelling of the combined data at 11 and 14 years, separately in boys and girls. In boys, the median ages at reporting growth spurt, body hair, skin changes, voice breaking and facial hair were 12.4, 13.0, 13.8, 13.8 and 14.8 years, respectively. In girls, the median ages at reporting growth spurt, breast development, body hair and skin changes were 11.2, 11.5, 11.7 and 12.6 years, respectively. Next, for each individual, each puberty trait was categorized into: (i) earlier puberty—if puberty was reported and the age at that response was <median age or (ii) later puberty—if pre-puberty was reported and age at response was ≥median age. Many responses were uninformative and thus excluded: if puberty was reported but age at response was >median age; or if pre-puberty was reported but age at response was <median age (46.2% for growth spurt, 66.8% for body hair, 58.7% for skin changes, 68.2% for voice breaking, 69.7% for facial hair, 49.1% breast development). For age at menarche, we took the first informative value across the three time points and categorized these into approximate tertiles: (i) 8–12 years, (ii) >12–13 years and (iii) >13 years.

### Potential confounders

Potential confounders were selected as those reportedly associated with the child’s physical activity and puberty timing.^42^ These included the most contemporary (i.e. closest to age 7 years or MCS4) self-reported maternal characteristics [i.e. age at delivery (MCS1–MCS2), active smoking during pregnancy (MCS1), alcohol consumption during pregnancy (MCS1), highest academic qualification level (MCS1–MCS4) and pre-pregnancy BMI (MCS1)], family income (MCS1–MCS4) and child characteristics [i.e. ethnicity (MCS1–MCS6), birthweight (MCS1–MCS2), gestational age (MCS1), breastfeeding duration (MCS1–MCS2), mental health (MCS3–MCS4), dietary behaviour (whether eating a variety of foods) (MCS4), regular sleep time (MCS3–MCS4), long-term health status (MCS3–MCS4) and BMI-for-age z score at 7 years (MCS4)]. Specifically, pre-pregnancy BMI was based on self-report or calculated by dividing the self-reported pre-pregnancy weight in kilograms by the squared height in metres. Family income was calculated by dividing the self-reported total net weekly household income by the number of household members according to assigned weights (i.e. 1 for first adult, 0.5 for each additional adult and 0.3 for each child under 14 years) on the Organisation for Economic Co-operation and Development’s equivalized income scale^43^ and converted into quintiles. The child’s mental health status was assessed using the Strength and Difficulties Questionnaire^44^ and classified as normal (0–13) or borderline to abnormal (14–40) based on total difficulties scores from four domains (peer problems, conduct disorders, hyperactivity and emotional problems).^45^ The child’s weight and height were measured by trained staff using electronic Tanita scales (Tanita UK Ltd, Middlesex, UK) and a Leicester Stadiometers (Seca Ltd, Birmingham, UK), respectively, and subsequently converted into BMI-for-age z scores based on the World Health Organization growth references.^46^

### Statistical analyses

The analysis sample included children with data for both physical activity and timing of any puberty trait, and who were from a singleton pregnancy and mother’s age at delivery of ≥18 years (Supplementary Figure S2, available as Supplementary data at *IJE* online). The excluded and included samples were compared using chi-squared tests for categorical variables and *t*-tests for continuous variables.

To investigate the associations of total daily counts (as a single exposure) with puberty timing, multivariable binary logistic [earlier vs later (reference) puberty], multinomial logistic [Tertile 1 and Tertile 3 vs Tertile 2 (reference)] and linear (for age at menarche) regression models were computed, with adjustment for potential confounders, separately in boys and girls. These models were further adjusted to assess the role of BMI-for-age z score at 11 years as a potential mediator.

Similar multivariable binary logistic, multinomial logistic and linear regression models were performed to examine the associations between physical activity intensity (fractions of total daily counts contributed by each intensity) and puberty timing, adjusted for the same potential confounders and mediator as above. In compositional models^47^^,^^48^ (Supplementary Text, available as Supplementary data at *IJE* online), the combined relative effects of physical activity intensities were tested by including isometrically log-ratio transformed fractions of counts from three intensities (light, moderate, vigorous) and empirical total daily accelerometer counts in the same model. The proportion of movement from sedentary was excluded as it did not contribute meaningfully to movement volume (Table 1). As a sensitivity test, we also conducted isomovement substitution analysis (Supplementary Text, available as Supplementary data at *IJE* online), which estimates the effect of the reallocation of movement (per 10%) from light to moderate or vigorous intensities, without changing total daily movement. Additionally, to understand the role of sedentary behaviour, we performed isotemporal substitution analysis (Supplementary Text, available as Supplementary data at *IJE* online) to estimate the effects of the reallocation of time spent (per 10 min) from sedentary behaviour to light, moderate or vigorous intensities.

**Table 1:** Comparisons of physical activity, puberty timing and body mass index between boys and girls

	Boys (*n = *2531)	Girls (*n = *3079)	*P*
**Physical activity based on accelerometers**	**Mean±SD**	**Mean±SD**	
Age at measurement, years	7.23±0.25	7.22±0.25	0.661
Spring	–0.36±0.58	–0.38±0.57	0.214
Winter	–0.15±0.72	–0.14±0.71	0.610
Number of valid measure days	5.7±1.5	5.5±1.6	<0.001
Proportion of weekdays for measure, %	77.8±13.5	78.5±14.1	0.045
Daily total time for measure, min	740.5±61.6	732.8±60.9	<0.001
Sedentary activity	388.1±65.1	398.7±67.8	<0.001
Light activity	283.3±40.9	278.6±41.1	<0.001
Moderate activity	46.8±13.2	37.9±11.5	<0.001
Vigorous activity	22.3±11.4	17.6±9.4	<0.001
Total daily counts, 100 000 per day	4.70±1.13	4.15±1.03	<0.001
Fraction of total counts from, %			
Sedentary activity	3.0E^–20^±1.1E^–21^	3.5E^–20^±2.1E^–20^	<0.001
Light activity	41.6±8.4	46.4±8.6	<0.001
Moderate activity	33.7±4.1	31.2±4.1	<0.001
Vigorous activity	24.6±8.0	22.4±7.3	<0.001
**Physical activity based on timesheet**	***n* (%)**	***n* (%)**	
Daily duration spent swimming, min			0.722
0	1372 (54.2)	1695 (55.1)	
1–29	1079 (42.6)	1281 (41.6)	
≥30	80 (3.2)	103 (3.4)	
Duration spent cycling, min			0.339
0	1632 (64.5)	2043 (66.4)	
1–29	763 (30.2)	881 (28.6)	
≥30	136 (5.4)	155 (5.0)	
A typical week for physical activity			0.288
No	660 (34.0)	831 (35.6)	
Yes	1279 (66.0)	1503 (64.4)	
**Puberty timing**	***n* (%)**	***n* (%)**	
Growth spurt			
Earlier (boys: <12.4; girls: <11.2 years)	847 (53.3)	563 (37.5)	
Later (boys: ≥12.4; girls: ≥11.2 years)	741 (46.7)	940 (62.5)	
Body hair growth			
Earlier (boys: <13.0; girls: <11.7 years)	235 (29.8)	995 (79.9)	
Later (boys: ≥13.0; girls: ≥11.7 years)	553 (70.2)	251 (20.1)	
Skin changes			
Earlier (boys: <13.8; girls: <12.6 years)	270 (21.8)	714 (51.9)	
Later (boys: ≥13.8; girls: ≥12.6 years)	969 (78.2)	662 (48.1)	
Voice breaking			
Earlier (boys: <13.8 years)	138 (13.3)	–	
Later (boys: ≥13.8 years)	900 (86.7)	–	
Facial hair growth			
Earlier (boys: <14.8 years)	863 (95.0)	–	
Later (boys: ≥14.8 years)	45 (5.0)	–	
Breast development			
Earlier (girls: <11.5 years)	–	916 (59.1)	
Later (girls: ≥11.5 years)	–	634 (40.9)	
Age at menarche			
Tertile 1 (8–12 years)	–	1515 (52.2)	
Tertile 2 (>12–13 years)	–	955 (32.9)	
Tertile 3 (>13–17 years)	–	434 (14.9)	
Age at menarche, years (mean±SD)	–	12.4±1.3	
**Weight status based on body mass index z score**	***n* (%)**	***n* (%)**	
At 7 years			0.287
Underweight (<–2 z score)	28 (1.1)	22 (0.7)	
Normal weight (≥2 to ≤1 z score)	1850 (73.5)	2275 (74.0)	
Overweight (>1 z score)	640 (25.4)	777 (25.3)	
At 11 years			0.193
Underweight (<–2 z score)	31 (91.3)	50 (1.7)	
Normal weight (≥2 to ≤1 z score)	1579 (64.6)	1954 (66.0)	
Overweight (>1 z score)	834 (34.1)	957 (32.3)	
**Body mass index, kg/m^2^**	**Mean±SD**	**Mean±SD**	
At 7 years	16.4±2.1	16.5±2.1	0.260
At 11 years	18.7±3.4	19.1±3.5	<0.001

Given the extent of missing data on covariates (missing *n = *1–496, 0.02–8.8%; at least one missing *n = *741, 13.2%), we used multiple imputation by chained equations with 50 imputed data sets^49^ to impute missing data on potential confounders and mediator under the assumption of missingness at random.

All statistical analyses were performed using Stata 15.1 (StataCorp. 2017. Stata Statistical Software: Release 15. College Station, TX: StataCorp LLC).

## Results

### Children’s characteristics

Among 18 314 singleton children born to adult mothers (Supplementary Figure S2, available as Supplementary data at *IJE* online), this study included 5610 children, comprising 2531 boys and 3079 girls. Compared with mothers of excluded children (*n = *12 704), mothers of the included children were more likely to be older and have higher family income and higher education level (Supplementary Table S1, available as Supplementary data at *IJE* online). Included children were more likely to be girls, White and heavier at birth but lighter at 7 and 11 years (Supplementary Table S1, available as Supplementary data at *IJE* online).

Among the included children, the mean total daily counts were 439 768 (SD = 111 278) counts, ranging from 68 380 to 1 555 514 counts, whereas the mean daily time spent in sedentary behaviour, and light, moderate and vigorous intensities were 394 (SD = 67), 281 (SD = 41), 42 (SD = 13) and 20 (SD = 11) min, respectively. More than half did not swim (*n = *3067, 54.7%) or cycle (*n = *3675, 65.5%) during the monitoring period, whereas others spent 1–29 min (*n = *2360, 42.1%; *n = *1644, 29.3%) or ≥30 min (*n = *183, 3.3%; *n = *21, 5.2%) per day swimming or cycling. The physical activity measured was mainly reported to be typical (*n = *2782, 65.1%) among those who responded (*n = *4273). Table 1 summarizes physical activity, puberty timing and BMI variables. Compared with boys, girls were less active at 7 years and had fewer days of valid physical activity data, more physical activity data on weekdays, shorter daily total monitor wear time and higher BMI at 11 years.

Supplementary Table S2 (available as Supplementary data at *IJE* online) shows the correlations between physical activity measures. In both boys and girls, total daily activity counts were strongly negatively correlated with fractions of total counts from sedentary and light intensities. Also, the fraction of total counts from vigorous activity was strongly negatively correlated with that from light intensity. Conversely, time spent in moderate and vigorous intensities was moderately positively correlated.

Supplementary Table S3 (available as Supplementary data at *IJE* online) shows the correlations between puberty timing. In boys, the timing of growth spurts was moderately correlated with timings of body hair, skin changes and voice breaking. In girls, the timing of growth spurts was moderately to strongly correlated with timings of hair, skin and breast changes.

### Physical activity and puberty timing

Table 2 shows the associations of total daily activity counts with puberty timing. In single exposure models, in boys, higher total counts were associated with lower risks for earlier (vs later) skin changes and voice breaking. In girls, higher total counts were associated with lower risks for earlier growth spurt, body hair growth, skin changes and menarche.

**Table 2:** Associations of physical activity with earlier (vs later) puberty timing in single exposure and compositional models

(a) Boys										
Model	Earlier growth spurt (*n = *1588)		Earlier body hair growth (*n = *788)		Earlier skin changes (*n = *1239)		Earlier facial hair (*n = *908)		Earlier voice breaking (*n = *1038)	
	OR (95% CI)	*P*	OR (95% CI)	*P*	OR (95% CI)	*P*	OR (95% CI)	*P*	OR (95% CI)	*P*
Single exposure model										
Total counts (per 100 000)	0.92 (0.83, 1.01)	0.085	0.93 (0.79, 1.10)	0.397	0.86 (0.75, 0.98)	0.022	1.22 (0.87, 1.70)	0.249	0.80 (0.66, 0.96)	0.018
Compositional model										
Total counts (per 100 000)	0.86 (0.73, 1.01)	0.065	0.76 (0.58, 1.00)	0.052	0.73 (0.58, 0.91)	0.006	1.14 (0.68, 1.91)	0.631	0.66 (0.49, 0.90)	0.010
Log-fraction of counts										
Light intensity	0.27 (0.05, 1.36)	0.112	0.40 (0.03, 5.71)	0.501	0.30 (0.03, 2.55)	0.269	0.53 (0.01, 108.04)	0.813	0.12 (0.01, 2.51)	0.173
Moderate intensity	0.50 (0.15, 1.70)	0.268	0.66 (0.09, 4.76)	0.679	1.18 (0.23, 5.99)	0.838	3.89 (0.12, 130.89)	0.449	0.27 (0.03, 2.21)	0.224
Vigorous intensity	0.61 (0.28, 1.35)	0.221	1.41 (0.38, 5.20)	0.610	0.88 (0.32, 2.44)	0.803	0.77 (0.08, 7.58)	0.824	0.66 (0.16, 2.79)	0.571

(b) Girls														
Model	Earlier growth spurt (*n = *1503)		Earlier body hair growth (*n = *1246)		Earlier skin changes (*n = *1376)		Earlier breast development (*n = *1550)		Earlier menarche (T1 vs T2)		Later menarche (T3 vs T2)		Age at menarche (*n = *2904)	
	OR (95% CI)	*P*	OR (95% CI)	*P*	OR (95% CI)	*P*	OR (95% CI)	*P*	OR (95% CI)	*P*	OR (95% CI)	*P*	β (95% CI)	*P*
Single exposure model														
Total counts (per 100 000)	0.85 (0.76, 0.96)	0.008	0.80 (0.68, 0.94)	0.005	0.80 (0.71, 0.90)	2.6E–4	0.91 (0.81, 1.02)	0.106	0.87 (0.80, 0.95)	0.002	0.91 (0.81, 1.02)	0.111	0.03 (–0.01, 0.08)	0.156
Compositional model														
Total counts (per 100 000)	0.90 (0.75, 1.08)	0.261	0.73 (0.57, 0.94)	0.014	0.76 (0.62, 0.92)	0.005	0.88 (0.73, 1.06)	0.188	0.81 (0.70, 0.93)	0.004	0.84 (0.68, 1.02)	0.080	0.04 (–0.03, 0.12)	0.272
Log-fraction of counts														
Light intensity	1.45 (0.22, 9.55)	0.702	0.38 (0.02, 6.08)	0.495	1.24 (0.15, 10.46)	0.842	0.39 (0.05, 2.75)	0.343	0.36 (0.08, 1.59)	0.179	0.92 (0.11, 7.97)	0.939	0.26 (–0.53, 1.05)	0.518
Moderate intensity	0.66 (0.21, 2.08)	0.477	0.79 (0.14, 4.57)	0.796	1.50 (0.40, 5.57)	0.549	2.00 (0.63, 6.36)	0.238	1.13 (0.48, 2.64)	0.785	1.49 (0.42, 5.37)	0.538	–0.14 (–0.60, 0.32)	0.560
Vigorous intensity	1.09 (0.50, 2.39)	0.828	0.85 (0.26, 2.79)	0.784	1.32 (0.55, 3.16)	0.533	0.52 (0.23, 1.20)	0.127	0.71 (0.39, 1.30)	0.264	1.22 (0.49, 3.01)	0.673	0.16 (–0.16, 0.48)	0.330

Adjusted for maternal characteristics (age, active smoking during pregnancy, alcohol consumption during pregnancy, education, pre-pregnancy body mass index, Organisation for Economic Co-operation and Development equivalized family income) and child characteristics (ethnicity, birthweight, gestational age, breastfeeding duration, mental health, dietary behaviour, regular sleep time, long-term health status and body-mass-index-for-age z score at 7 years).

OR, odds ratio.

No physical activity intensity was associated with lower risk for earlier puberty timing in compositional (Table 2) or isomovement substitution analyses (Supplementary Table S4, available as Supplementary data at *IJE* online). In isotemporal substitution analyses (Supplementary Table S4, available as Supplementary data at *IJE* online) among girls, reallocation of time spent from sedentary behaviour to light-intensity activity was associated with lower risk for earlier skin changes and reallocation from sedentary to vigorous intensity was associated with lower risk for earlier breast development.

All observed associations between physical activity, particularly total daily activity counts, and puberty timing were unattenuated on further adjustment for BMI at 11 years (Supplementary Table S5, available as Supplementary data at *IJE* online).

## Discussion

Using accelerometry measures of physical activity in this prospective British cohort study, we demonstrated associations of pre-pubertal total movement volume with puberty timing in boys and girls. Higher total daily counts were associated with lower risks for earlier timings of puberty progression (i.e. voice breaking and menarche, respectively) and other puberty traits (growth spurt, body hair growth and skin changes) in boys and girls. All observed associations for puberty timing persisted on additional adjustment for BMI during puberty at 11 years. Beyond total movement volume, no specific intensity of physical activity was associated with puberty timing, but reallocation of time spent from sedentary to light or vigorous activity was associated with lower risks of earlier puberty in girls.

The present study substantially extends existing evidence^22–26^ by analysing more robust and comprehensive data on physical activity in both boys and girls, with a longer duration of follow-up from 7 to 17 years and in a larger sample size. Previous studies assessed overall physical activity duration and energy expenditure using parent- or self-reported questionnaires among pre-menarcheal girls at 6–18 years and followed up menarche status only 1–3 years later (*n = *167–2487).^22–26^ By contrast, we included objective accelerometry measures at 7 years (i.e. before the physiological age range for puberty onset), which reduced exposure measurement errors, minimized the possibility of reverse causality and allowed exploration of the potential role of specific physical activity intensities. Also, we examined age at menarche and timings of other puberty traits in boys and girls that were recorded repeatedly during adolescence until young adulthood. Our findings show a high degree of internal validity with adjustment for a wide range of potential confounders including dietary behaviour and BMI at baseline, which were not considered in most previous longitudinal cohorts^22–26^ or case–control studies of athletes.^20^ Among previous prospective cohorts,^22–26^ only one reported an association of higher overall physical activity and lower risk for earlier menarche in girls.^22^ Similarly, the only previous randomized–controlled trial estimated that the effect of a multicomponent intervention on reaching menarche within the 2-year study period could be partially explained by change in physical activity.^21^

Previous single exposure analyses on the association between physical activity and later BMI in the same cohort showed that more time spent in moderate-to-vigorous intensity, but not sedentary activity or total counts, was associated with a lower percentage change in BMI at 11 years only in boys.^19^ Our findings add that higher overall physical activity, but not any specific intensity level, was associated with lower risks for earlier timings of several puberty traits in girls and boys, independently of any effects of physical activity on conditional change in BMI. Together, these findings indicate a role of moderate-to-vigorous-intensity physical activity on reducing conditional change in BMI, in contrast to the role of total movement on reducing the risk of early puberty timing, regardless of intensity. Hence, our findings suggest that there may be other adiposity-independent underlying mechanisms linking physical activity to puberty timing. It is possible that differences in body composition, independently of BMI, alter the amount of leptin released from adipose tissue,^50^ which then acts on the reproductive hormone axis^51^ or gonadotropin-releasing hormone pulsatility.^52^ It is also possible that a higher total volume of physical activity promotes muscle mass gain, which increases glucose transport isoforms GLUT4 to absorb blood glucose^53^ and reduce insulin resistance, in turn decreasing the bioavailability of sex steroids and delaying pubertal development in boys and girls.^1^ Furthermore, the present study highlights that increasing daily total physical activity by 100 000 counts (i.e. nearly 1 SD) may reduce the relative risk of earlier pubertal development by 13–20%. This risk reduction is similar to that for total energy and protein intakes (20–30%),^18^ slightly higher than that for endocrine-disrupting chemicals (10%)^54^ and potentially might be additive to the effects of obesity prevention (50%).^14^

The present findings need cautious interpretation due to several limitations. There are modest differences in maternal and child characteristics between the included and excluded children, but these factors were adjusted for in all models to reduce potential selection bias.^55^ However, the differences may still limit the generalizability of our findings in particular to populations with lower family income and education, and non-White ethnicity. Furthermore, non-wear time was based on a short period (20 min) of consecutive zero-counts, which may have underestimated the time spent in sedentary activity. Given the limitations of accelerometers, vertical movement of the trunk such as cycling (due to wearing at hip), aquatic activities such as swimming (due to non-waterproofing) and high-intensity activities (due to longer epochs) could not be well captured, which may have underestimated physical activity overall and by intensity; however, only a few included in this study reported having spent >30 min daily swimming or cycling during the monitoring period. Although the average movement intensity for each physical activity intensity category was estimated, the estimated sum of the counts was nearly perfectly correlated with the empirical total counts. We also minimized the potential bias due to estimation errors of counts for each physical activity intensity level by adjusting our analyses for the empirical total activity counts instead of the estimated total sum of counts.

We also acknowledge other limitations that limit the ability to make conclusions on pubertal outcomes beyond the age at menarche in girls. Pubertal outcomes were reported by parents and children at different ages, which did not allow direct evaluation of the agreement in responses by parents and children at the same age. Nonetheless, we observed only a small proportion (<6%) of inconsistent reporting (i.e. puberty at one visit but pre-puberty at the next) for most physical pubertal traits based on those with both responses. Any disagreement in responses between parents and children would be expected to lead to random misclassification of puberty status and bias findings towards the null, unless parents of physically active children (or the children themselves) were less observant about their child’s (or their own) pubertal development than parents of sedentary children. Although the pubertal development questionnaire was not validated against Tanner staging,^2^^,^^3^ the question on age at menarche in girls has been well validated.^56^ There was no objective measure of puberty timing such as age at peak height velocity, since height was not regularly measured. However, we obtained consistent findings for earlier pubertal development indicated by several traits and earlier menarche (from the established and validated assessment of age at menarche) in girls. Compared with a contemporary UK cohort [i.e. Avon Longitudinal Study of Parents and Children (ALSPAC)], which assessed pubertal development by annual parent- or self-reports at 8–17 years,^17^^,^^18^ we observed similar estimated mean or median ages at menarche (12.4 vs 12.7 years) in girls and voice breaking (13.8 vs 13.6 years) in boys. However, median ages at physical puberty traits were later in MCS than in ALSPAC^17^^,^^18^ (body vs pubic hair growth: 11.3 vs 11.0 years in boys and 11.7 vs 10.8 years in girls) especially in breast development in girls (11.5 vs 10.0 years), possibly due to the nature of pubertal assessment in MCS including only two time points, later age at first reporting and crude reporting of puberty status (‘has definitely started’) rather than the first evidence of puberty (Tanner stage 2) by annual parent- or self-reports at 8–17 years in ALSPAC (albeit not by gold standard trained clinical examination). Also, the earlier median age at growth spurt in MCS than in ALSPAC^17^^,^^18^ (12.4 vs 13.6 years in boys and 11.2 vs 11.7 years in girls) may reflect differences in subjective (‘has definitely started’) and objective assessment (peak height velocity based on longitudinal height measures). Hence, we dichotomized puberty status based on median ages to reduce uncertainty in variation between individuals. Although we were unable to classify timings of puberty traits in many children due to the wide range of ages at each assessment time point, there were only modest differences in the ages at questionnaire completion at 11 and 14 years between those with and without classifiable puberty status for each trait (Supplementary Table S6, available as Supplementary data at *IJE* online). Our method of dichotomizing puberty status also led to a small sample in the category ‘later facial hair in boys’ and thus findings for this trait may be inconclusive rather than null. Moreover, the associations between physical activity and puberty timing in boys may be considered to be weak in view of the multiple outcomes tested, although the effect estimates were similar to those seen in girls. Our observations may be affected by residual confounding due to unmeasured factors such as specific dietary factors^57^ including intakes of total energy, total protein^18^ and polyunsaturated fatty acids,^17^ and neighbourhood environment.^58^^,^^59^ We analysed BMI at 11 years as a potential mediator; however, puberty may have occurred before 11 years in some children and hence the inclusion of BMI at 11 years might introduce over-adjustment.

In conclusion, in this first prospective analysis of objectively measured physical activity, we showed that higher total movement volume, but not any intensity of physical activity, was associated with lower risks for earlier timing of several puberty traits, particularly in girls. These associations were independent of baseline and change in BMI. Our findings suggest that promoting overall physical activity, by any intensity or by reducing time spent in sedentary behaviour, may be a potential strategy to avoid early puberty timing, especially in girls.

## Ethics approval

The study was approved by the South West Multi-Centre Research Ethics Committee, the London Multi-Centre Research Ethics Committee, the Northern and the Yorkshire Research Ethics Committee, the Yorkshire and Humber Research Ethics Committee, the National Research Ethics Service Research Ethics Committee London—Central and the National Research Ethics Service Research Ethics Committee North East—York. Parents and children provided their written informed consent.

## Supplementary Material

dyad063_Supplementary_Data

## Data Availability

The MCS data including data sets analysed in the present study are freely available to bona fide researchers under standard access conditions via the UK Data Service (http://ukdataservice.ac.uk) and the MCS website provides detailed information on the study (http://www.cls.ioe.ac.uk/mcs).
