# Supplementary material for: Pre-pubertal accelerometer-assessed physical activity and timing of puberty in British boys and girls: the Millennium Cohort Study
Source: Int J Epidemiol. 2023 May 19;52(5):1316–27. doi: 10.1093/ije/dyad063 (PMC10555885; doi:10.1093/ije/dyad063)
Supplement: dyad063_Supplementary_Data [file dyad063_supplementary_data.docx]

**Supplementary Figure S1** Flow chart for derivation of fractions of total daily counts contributed by individual physical activity intensities

Calculate the fractions of total counts contributed by each intensity (%) across all valid days in each individual

Standardise the derived variables by adjusting for covariates

Calculate the fractions of total counts contributed by each intensity (%) for each valid day in each individual

Estimate the average intensity (counts per minute) for each physical activity intensity level

Daily summary data among children with at least 10 hours of valid wear data per day for at least two days

**Supplementary Figure S2** Study inclusion criteria

19500 children

- Twins (n=506)
- Triplets (n=15)

Unknown ethnic background (n=87)

18979 singletons

18892 singletons

From teenage pregnancy (n=578)

Missing physical activity measures (n=11926)

18314 children

Missing any puberty trait (n=778)

5610 children in final analytical sample

6388 children

**Supplementary Text** Statistical models with physical activity measures as exposures

Compositional movement analysis:

*Outcome = Total daily counts + Light-PA_ILR_ + Moderate-PA_ILR_ + Vigorous-PA_ILR_*

PA_ILR_, isometric log-ratio of the fraction of total daily counts contributed by each intensity

Movement contributed by sedentary activity is ignored as values are very close to zero

Beta values estimate the effect on the outcome of the fraction that each intensity contributes to total daily movement

Isomovement substitution analysis:

*Outcome = Total daily counts +* *Moderate-PA + Vigorous-PA*

PA, fraction of total daily movement contributed by each intensity

Beta values estimate the effect on the outcome of substituting each intensity for light physical activity, without changing total daily movement

Isotemporal substitution analysis:

*Outcome = Time_light_ + Time_moderate_ + Time_vigorous_*

Beta values estimate the effect on the outcome of the time spent in each intensity

| **Supplementary Table S1** Comparisons of characteristics between excluded and included children | | | |
| --- | --- | --- | --- |
|  | Excluded (n=12704) | Included (n=5610) | P value |
| **Maternal characteristics** |  |  |  |
| Education (n, %) |  |  | <0.001 |
| None/other | 3329 (26.3) | 715 (12.8) |  |
| GCSE D-G | 1425 (11.3) | 494 (8.8) |  |
| GCSE A-C | 4295 (34.0) | 1802 (32.2) |  |
| A-level/diploma | 2042 (16.1) | 1235 (22.1) |  |
| First/higher degree | 1557 (12.3) | 1356 (24.2) |  |
| OECD equivalized family income (n, %) |  |  | <0.001 |
| Q1 | 2965 (23.4) | 548 (9.8) |  |
| Q2 | 2763 (21.8) | 841 (15.0) |  |
| Q3 | 2546 (20.1) | 1135 (20.3) |  |
| Q4 | 2309 (18.2) | 1411 (25.2) |  |
| Q5 | 2077 (16.4) | 1671 (29.8) |  |
| Age at delivery, years | 28.0±5.8 | 29.9±5.3 | <0.001 |
| Pre-pregnancy body mass index, kg/m^2^ | 23.7±4.5 | 23.8±4.3 | 0.074 |
|  |  |  |  |
| **Child characteristics** |  |  |  |
| Sex (n, %) |  |  | <0.001 |
| Boys | 6878 (54.1) | 2531 (45.1) |  |
| Girls | 5826 (45.9) | 3079 (54.9) |  |
| Ethnicity (n, %) |  |  | <0.001 |
| White | 10098 (79.5) | 4915 (87.6) |  |
| Asian | 1620 (12.8) | 408 (7.3) |  |
| Black | 570 (4.5) | 129 (2.3) |  |
| Mixed | 416 (3.3) | 158 (2.8) |  |
| Birth weight, kg | 3.34±0.59 | 3.40±0.56 | <0.001 |
| Body mass index at 7 years, kg/m^2^ | 16.8±2.5 | 16.4±2.1 | <0.001 |
| Body-mass-index-for-age z scores at 7 years | 0.58±1.23 | 0.41±1.09 | <0.001 |
| Body mass index at 11 years, kg/m^2^ | 19.5±3.8 | 18.9±3.4 | <0.001 |
| Body-mass-index-for-age z scores at 11 years | 0.66±1.28 | 0.48±1.19 | <0.001 |

| **Supplementary Table S2** Pearson’s correlations between physical activity measures at 7 years | | | | | |
| --- | --- | --- | --- | --- | --- |
|  | Sedentary activity | Light intensity | Moderate intensity | Vigorous intensity | Total daily counts |
| Boys |  |  |  |  |  |
| Fraction of total counts from, % |  |  |  |  |  |
| Sedentary activity | 1.00 |  |  |  |  |
| Light intensity | 0.66 | 1.00 |  |  |  |
| Moderate intensity | -0.29 | -0.28 | 1.00 |  |  |
| Vigorous intensity | -0.53 | -0.88 | -0.22 | 1.00 |  |
| Total daily counts | -0.85 | -0.76 | 0.23 | 0.66 | 1.00 |
| Time spent in, minutes |  |  |  |  |  |
| Sedentary activity | 1.00 |  |  |  |  |
| Light intensity | -0.37 | 1.00 |  |  |  |
| Moderate intensity | -0.44 | 0.41 | 1.00 |  |  |
| Vigorous intensity | -0.29 | 0.09 | 0.67 | 1.00 |  |
|  |  |  |  |  |  |
| Girls |  |  |  |  |  |
| Fraction of total counts from, % |  |  |  |  |  |
| Sedentary activity | 1.00 |  |  |  |  |
| Light intensity | 0.63 | 1.00 |  |  |  |
| Moderate intensity | -0.41 | -0.46 | 1.00 |  |  |
| Vigorous intensity | -0.49 | -0.87 | -0.02 | 1.00 |  |
| Total daily counts | -0.81 | -0.74 | 0.37 | 0.64 | 1.00 |
| Time spent in, minutes |  |  |  |  |  |
| Sedentary activity | 1.00 |  |  |  |  |
| Light intensity | -0.41 | 1.00 |  |  |  |
| Moderate intensity | -0.50 | 0.46 | 1.00 |  |  |
| Vigorous intensity | -0.35 | 0.14 | 0.70 | 1.00 |  |

| **Supplementary Table S3** Spearman’s correlations between puberty timing | | | | | |  |  |  |
| --- | --- | --- | --- | --- | --- | --- | --- | --- |
|  | Growth spurt | Body hair growth | Skin changes | Voice breaking | Facial hair growth | Breast development | Categorical age at menarche | Age at menarche |
| Boys |  |  |  |  |  |  |  |  |
| Growth spurt | 1.00 |  |  |  |  |  |  |  |
| Body hair growth | 0.64 | 1.00 |  |  |  |  |  |  |
| Skin changes | 0.51 | 0.73 | 1.00 |  |  |  |  |  |
| Voice breaking | 0.39 | 0.58 | 0.85 | 1.00 |  |  |  |  |
| Facial hair growth | 0.13 | 0.21 | 0.15 | 0.20 | 1.00 |  |  |  |
|  |  |  |  |  |  |  |  |  |
| Girls |  |  |  |  |  |  |  |  |
| Growth spurt | 1.00 |  |  |  |  |  |  |  |
| Body hair growth | 0.49 | 1.00 |  |  |  |  |  |  |
| Skin changes | 0.47 | 0.63 | 1.00 |  |  |  |  |  |
| Breast development | 0.60 | 0.67 | 0.56 |  |  | 1.00 |  |  |
| Age at menarche | 0.36 | 0.43 | 0.43 |  |  | 0.51 | 0.94 | 1.00 |

| **Supplementary Table S4** Associations of physical activity with earlier (vs. later) puberty timing in isomovement and isotemporal substitution models | | | | | | | | | | | | | | | | |  |  |
| --- | --- | --- | --- | --- | --- | --- | --- | --- | --- | --- | --- | --- | --- | --- | --- | --- | --- | --- |
|  | OR (95% CI) | P value |  | OR (95% CI) | P value |  | OR (95% CI) | P value |  | OR (95% CI) | P value |  | OR (95% CI) | P value | OR (95% CI) | P value | β (95% CI) | P value |
| **Boys** | Earlier growth spurt  (n=1588) | |  | Earlier body hair growth (n=788) | |  | Earlier skin changes (n=1239) | |  | Earlier facial hair  (n=908) | |  | Earlier voice breaking (n=1038) | |  |  |  |  |
| Isomovement substitution |  |  |  |  |  |  |  |  |  |  |  |  |  |  |  |  |  |  |
| Total counts (per 100000) | 0.87 (0.74, 1.01) | 0.073 |  | 0.75 (0.57, 0.98) | 0.038 |  | 0.73 (0.59, 0.92) | 0.006 |  | 1.15 (0.70, 1.91) | 0.577 |  | 0.69 (0.51, 0.93) | 0.015 |  |  |  |  |
| Fractions of counts (per 10%) |  |  |  |  |  |  |  |  |  |  |  |  |  |  |  |  |  |  |
| Moderate intensity | 1.07 (0.79, 1.44) | 0.665 |  | 1.13 (0.71, 1.80) | 0.612 |  | 1.38 (0.91, 2.08) | 0.131 |  | 1.67 (0.70, 4.02) | 0.251 |  | 1.00 (0.58, 1.71) | 0.996 |  |  |  |  |
| Vigorous intensity | 1.11 (0.90, 1.37) | 0.343 |  | 1.47 (1.03, 2.09) | 0.033 |  | 1.26 (0.94, 1.69) | 0.116 |  | 1.01 (0.53, 1.93) | 0.967 |  | 1.32 (0.90, 1.96) | 0.157 |  |  |  |  |
| Isotemporal substitution |  |  |  |  |  |  |  |  |  |  |  |  |  |  |  |  |  |  |
| Time spent (per 10 minutes) |  |  |  |  |  |  |  |  |  |  |  |  |  |  |  |  |  |  |
| Light intensity | 0.99 (0.96, 1.02) | 0.498 |  | 0.96 (0.91, 1.01) | 0.134 |  | 0.95 (0.91, 1.00) | 0.039 |  | 1.01 (0.92, 1.11) | 0.829 |  | 0.95 (0.90, 1.01) | 0.118 |  |  |  |  |
| Moderate intensity | 0.95 (0.84, 1.08) | 0.423 |  | 0.92 (0.75, 1.12) | 0.397 |  | 1.03 (0.87, 1.23) | 0.710 |  | 1.28 (0.85, 1.93) | 0.232 |  | 0.86 (0.68, 1.08) | 0.192 |  |  |  |  |
| Vigorous intensity | 0.99 (0.87, 1.12) | 0.850 |  | 1.08 (0.88, 1.33) | 0.468 |  | 0.87 (0.73, 1.05) | 0.149 |  | 0.93 (0.62, 1.41) | 0.735 |  | 1.01 (0.80, 1.28) | 0.903 |  |  |  |  |
|  |  |  |  |  |  |  |  |  |  |  |  |  |  |  |  |  |  |  |
| **Girls** | Earlier growth spurt  (n=1503) | |  | Earlier body hair growth (n=1246) | |  | Earlier skin changes (n=1376) | |  | Earlier breast development (n=1550) | |  | Earlier menarche  (T1 vs. T2) | | Later menarche  (T3 vs. T2) | | Age at menarche  (n=2904) | |
| Isomovement substitution |  |  |  |  |  |  |  |  |  |  |  |  |  |  |  |  |  |  |
| Total counts (per 100000) | 0.90 (0.75, 1.08) | 0.261 |  | 0.74 (0.58, 0.95) | 0.017 |  | 0.75 (0.62, 0.91) | 0.003 |  | 0.91 (0.76, 1.09) | 0.302 |  | 0.83 (0.72, 0.95) | 0.070 | 0.84 (0.69, 1.01) | 0.070 | 0.04 (-0.04, 0.11) | 0.338 |
| Fractions of counts (per 10%) |  |  |  |  |  |  |  |  |  |  |  |  |  |  |  |  |  |  |
| Moderate intensity | 0.80 (0.58, 1.10) | 0.162 |  | 1.09 (0.71, 1.68) | 0.696 |  | 1.11 (0.80, 1.55) | 0.523 |  | 1.36 (0.98, 1.89) | 0.063 |  | 1.19 (0.93, 1.51) | 0.159 | 1.24 (0.88, 1.74) | 0.217 | -0.05 (-0.18, 0.08) | 0.423 |
| Vigorous intensity | 0.96 (0.76, 1.19) | 0.688 |  | 1.13 (0.84, 1.52) | 0.420 |  | 1.10 (0.87, 1.38) | 0.441 |  | 0.90 (0.73, 1.12) | 0.360 |  | 1.05 (0.89, 1.24) | 0.577 | 1.09 (0.87, 1.38) | 0.454 | 0.01 (-0.08, 0.10) | 0.772 |
| Isotemporal substitution |  |  |  |  |  |  |  |  |  |  |  |  |  |  |  |  |  |  |
| Time spent (per 10 minutes) |  |  |  |  |  |  |  |  |  |  |  |  |  |  |  |  |  |  |
| Light intensity | 1.00 (0.97, 1.04) | 0.893 |  | 0.96 (0.92, 1.00) | 0.078 |  | 0.96 (0.93, 0.99) | 0.022 |  | 0.98 (0.95, 1.02) | 0.275 |  | 0.97 (0.95, 1.00) | 0.054 | 0.97 (0.94, 1.01) | 0.135 | 0.01 (-0.01, 0.02) | 0.668 |
| Moderate intensity | 0.88 (0.75, 1.03) | 0.118 |  | 0.92 (0.75, 1.13) | 0.419 |  | 0.96 (0.82, 1.13) | 0.635 |  | 1.14 (0.97, 1.34) | 0.121 |  | 0.99 (0.87, 1.11) | 0.809 | 1.06 (0.90, 1.26) | 0.469 | 0.01 (-0.06, 0.07) | 0.811 |
| Vigorous intensity | 0.97 (0.82, 1.16) | 0.765 |  | 0.93 (0.73, 1.17) | 0.516 |  | 0.88 (0.73, 1.05) | 0.152 |  | 0.80 (0.66, 0.95) | 0.014 |  | 0.91 (0.80, 1.04) | 0.160 | 0.88 (0.73, 1.06) | 0.177 | 0.03 (-0.05, 0.10) | 0.476 |
| adjusted for maternal characteristics (age, active smoking during pregnancy, alcohol consumption during pregnancy, education, pre-pregnancy body mass index, OECD equivalized family income) and child characteristics (ethnicity, birth weight, gestational age, breastfeeding duration, mental health, dietary behavior, regular sleep time, long-term health status and body-mass-index-for-age z scores at 7 years | | | | | | | | | | | | | | | | | | |

| **Supplementary Table S5** Associations of physical activity with earlier (vs. later) puberty timing, on further adjustment for body mass index during puberty | | | | | | | | | | | | | | | | |  |  |
| --- | --- | --- | --- | --- | --- | --- | --- | --- | --- | --- | --- | --- | --- | --- | --- | --- | --- | --- |
|  | OR (95% CI) | P value |  | OR (95% CI) | P value |  | OR (95% CI) | P value |  | OR (95% CI) | P value |  | OR (95% CI) | P value | OR (95% CI) | P value | β (95% CI) | P value |
| **Boys** | Earlier growth spurt  (n=1588) | |  | Earlier body hair growth (n=788) | |  | Earlier skin changes (n=1239) | |  | Earlier facial hair  (n=908) | |  | Earlier voice breaking (n=1038) | |  |  |  |  |
| Single exposure model |  |  |  |  |  |  |  |  |  |  |  |  |  |  |  |  |  |  |
| Total counts (per 100000) | 0.92 (0.83, 1.02) | 0.097 |  | 0.93 (0.79, 1.10) | 0.389 |  | 0.85 (0.75, 0.98) | 0.021 |  | 1.22 (0.87, 1.70) | 0.241 |  | 0.80 (0.66, 0.96) | 0.018 |  |  |  |  |
| Compositional model |  |  |  |  |  |  |  |  |  |  |  |  |  |  |  |  |  |  |
| Total counts (per 100000) | 0.83 (0.71, 0.98) | 0.024 |  | 0.75 (0.57, 0.99) | 0.040 |  | 0.72 (0.57, 0.91) | 0.005 |  | 1.09 (0.65, 1.84) | 0.736 |  | 0.66 (0.48, 0.90) | 0.009 |  |  |  |  |
| Log-fraction of counts |  |  |  |  |  |  |  |  |  |  |  |  |  |  |  |  |  |  |
| Light intensity | 0.19 (0.04, 0.96) | 0.044 |  | 0.35 (0.02, 4.98) | 0.438 |  | 0.24 (0.03, 2.08) | 0.195 |  | 0.31 (0.01, 71.94) | 0.675 |  | 0.12 (0.01, 2.47) | 0.170 |  |  |  |  |
| Moderate intensity | 0.43 (0.13, 1.48) | 0.182 |  | 0.57 (0.08, 4.16) | 0.580 |  | 0.97 (0.19, 4.91) | 0.972 |  | 3.09 (0.09, 108.3) | 0.533 |  | 0.27 (0.03, 2.19) | 0.221 |  |  |  |  |
| Vigorous intensity | 0.58 (0.26, 1.29) | 0.180 |  | 1.39 (0.37, 5.16) | 0.623 |  | 0.82 0.30, 2.29) | 0.710 |  | 0.686 0.07, 6.86) | 0.748 |  | 0.66 (0.16, 2.78) | 0.567 |  |  |  |  |
| Isomovement substitution |  |  |  |  |  |  |  |  |  |  |  |  |  |  |  |  |  |  |
| Total counts (per 100000) | 0.84 (0.72, 0.98) | 0.031 |  | 0.74 (0.56, 0.97) | 0.030 |  | 0.73 (0.58, 0.91) | 0.005 |  | 1.12 (0.68, 1.86) | 0.655 |  | 0.69 (0.51, 0.93) | 0.015 |  |  |  |  |
| Fractions of counts (per 10%) |  |  |  |  |  |  |  |  |  |  |  |  |  |  |  |  |  |  |
| Moderate intensity | 1.10 (0.81, 1.49) | 0.546 |  | 1.11 (0.70, 1.78) | 0.657 |  | 1.35 (0.89, 2.05) | 0.159 |  | 1.71 (0.71, 4.10) | 0.232 |  | 1.00 (0.58, 1.71) | 0.996 |  |  |  |  |
| Vigorous intensity | 1.18 (0.95, 1.46) | 0.135 |  | 1.51 (1.06, 2.15) | 0.023 |  | 1.29 (0.96, 1.73) | 0.089 |  | 1.07 (0.56, 2.05) | 0.834 |  | 1.33 0.90, 1.96) | 0.156 |  |  |  |  |
| Isotemporal substitution |  |  |  |  |  |  |  |  |  |  |  |  |  |  |  |  |  |  |
| Time spent (per 10 minutes) |  |  |  |  |  |  |  |  |  |  |  |  |  |  |  |  |  |  |
| Light intensity | 0.98 (0.95, 1.01) | 0.219 |  | 0.96 (0.91, 1.01) | 0.122 |  | 0.95 (0.91, 1.00) | 0.041 |  | 1.00 (0.91, 1.10) | 0.985 |  | 0.95 (0.90, 1.01) | 0.119 |  |  |  |  |
| Moderate intensity | 0.95 (0.84, 1.08) | 0.415 |  | 0.90 (0.74, 1.10) | 0.307 |  | 1.02 (0.86, 1.21) | 0.841 |  | 1.28 (0.85, 1.93) | 0.235 |  | 0.86 (0.68, 1.08) | 0.188 |  |  |  |  |
| Vigorous intensity | 1.01 (0.88, 1.15) | 0.915 |  | 1.10 (0.89, 1.36) | 0.356 |  | 0.89 (0.74, 1.07) | 0.217 |  | 0.95 (0.63, 1.44) | 0.807 |  | 1.02 (0.80, 1.29) | 0.890 |  |  |  |  |
|  |  |  |  |  |  |  |  |  |  |  |  |  |  |  |  |  |  |  |
| **Girls** | Earlier growth spurt  (n=1503) | |  | Earlier body hair growth (n=1246) | |  | Earlier skin changes (n=1376) | |  | Earlier breast development (n=1550) | |  | Earlier menarche  (T1 vs. T2) | | Later menarche  (T3 vs. T2) | | Age at menarche  (n=2904) | |
| Single exposure model |  |  |  |  |  |  |  |  |  |  |  |  |  |  |  |  |  |  |
| Total counts (per 100000) | 0.85 (0.76, 0.96) | 0.010 |  | 0.79 (0.67, 0.93) | 0.004 |  | 0.79 (0.70, 0.89) | 1.7E-4 |  | 0.90 (0.80, 1.01) | 0.074 |  | 0.87 (0.80, 0.95) | 0.002 | 0.91 (0.81, 1.03) | 0.122 | 0.04 (-0.01, 0.08) | 0.116 |
| Compositional model |  |  |  |  |  |  |  |  |  |  |  |  |  |  |  |  |  |  |
| Total counts (per 100000) | 0.88 (0.73, 1.06) | 0.179 |  | 0.67 (0.52, 0.87) | 0.003 |  | 0.74 (0.61, 0.90) | 0.002 |  | 0.82 (0.67, 1.00) | 0.045 |  | 0.80 (0.69, 0.92) | 0.002 | 0.85 0.70, 1.04) | 0.122 | 0.06 (-0.01, 0.14) | 0.114 |
| Log-fraction of counts |  |  |  |  |  |  |  |  |  |  |  |  |  |  |  |  |  |  |
| Light intensity | 1.24 (0.18, 8.30) | 0.828 |  | 0.27 (0.02, 4.46) | 0.361 |  | 1.28 (0.15, 11.12) | 0.826 |  | 0.26 (0.03, 2.03) | 0.200 |  | 0.33 (0.08, 1.45) | 0.144 | 1.05 (0.12, 9.13) | 0.968 | 0.36 (-0.41, 1.13) | 0.361 |
| Moderate intensity | 0.61 (0.19, 1.93) | 0.397 |  | 0.72 (0.12, 4.25) | 0.718 |  | 1.54 (0.40, 5.889) | 0.528 |  | 1.92 (0.58, 6.36) | 0.283 |  | 1.04 (0.44, 2.44) | 0.936 | 1.71 (0.48, 6.07) | 0.403 | -0.04 (-0.49, 0.41) | 0.858 |
| Vigorous intensity | 1.13 (0.51, 2.50) | 0.761 |  | 0.92 (0.28, 3.03) | 0.885 |  | 1.43 (0.59, 3.48) | 0.425 |  | 0.53 (0.22, 1.26) | 0.152 |  | 0.72 (0.39, 1.31) | 0.280 | 1.18 (0.48, 2.93) | 0.722 | 0.14 (9-0.18, 0.45) | 0.392 |
| Isomovement substitution |  |  |  |  |  |  |  |  |  |  |  |  |  |  |  |  |  |  |
| Total counts (per 100000) | 0.88 (0.74, 1.06) | 0.174 |  | 0.68 (0.53, 0.88) | 0.003 |  | 0.73 (0.60, 0.88) | 0.001 |  | 0.85 (0.70, 1.02) | 0.083 |  | 0.82 (0.71, 0.94) | 0.004 | 0.85 (0.70, 1.04) | 0.110 | 0.05 (-0.02, 0.12) | 0.145 |
| Fractions of counts (per 10%) |  |  |  |  |  |  |  |  |  |  |  |  |  |  |  |  |  |  |
| Moderate intensity | 0.81 (0.58, 1.11) | 0.189 |  | 1.14 (0.73, 1.76) | 0.568 |  | 1.13 (0.81, 1.58) | 0.475 |  | 1.45 (1.03, 2.05) | 0.032 |  | 1.18 (0.93, 1.50) | 0.179 | 1.26 0.90, 1.78) | 0.180 | -0.05 9-0.17, 0.08) | 0.482 |
| Vigorous intensity | 1.01 (0.80, 1.26) | 0.961 |  | 1.26 (0.3, 1.71) | 0.133 |  | 1.14 (0.90, 1.44) | 0.283 |  | 0.99 (0.79, 1.24) | 0.918 |  | 1.07 (0.91, 1.27) | 0.404 | 1.05 (0.83, 1.33) | 0.690 | -0.02 (-0.11, 0.07) | 0.684 |
| Isotemporal substitution |  |  |  |  |  |  |  |  |  |  |  |  |  |  |  |  |  |  |
| Time spent (per 10 minutes) |  |  |  |  |  |  |  |  |  |  |  |  |  |  |  |  |  |  |
| Light intensity | 1.00 (0.96, 1.03) | 0.961 |  | 0.95 (0.90, 0.99) | 0.021 |  | 0.96 (0.92, 0.99) | 0.013 |  | 0.97 (0.93, 1.00) | 0.081 |  | 0.97 (0.95, 1.00) | 0.037 | 0.98 (0.94, 1.01) | 0.176 | 0.01 (-0.01, 0.02) | 0.406 |
| Moderate intensity | 0.88 (0.74, 1.03) | 0.106 |  | 0.90 (0.73, 1.12) | 0.347 |  | 0.95 (0.81, 1.13) | 0.570 |  | 1.14 (0.96, 1.35) | 0.142 |  | 0.98 (0.86, 1.10) | 0.687 | 1.09 (0.92, 1.29) | 0.335 | 0.02 (-0.04, 0.08) | 0.557 |
| Vigorous intensity | 1.00 (0.84, 1.19) | 0.966 |  | 0.97 (0.76, 1.23) | 0.789 |  | 0.89 (0.74, 1.06) | 0.187 |  | 0.81 (0.67, 0.98) | 0.032 |  | 0.92 (0.81, 1.05) | 0.236 | 0.85 (0.70, 1.03) | 0.095 | 0.01 (-0.06, 0.08) | 0.825 |
| adjusted for maternal characteristics (age, active smoking during pregnancy, alcohol consumption during pregnancy, education, pre-pregnancy body mass index, OECD equivalized family income) and child characteristics (ethnicity, birth weight, gestational age, breastfeeding duration, mental health, dietary behavior, regular sleep time, long-term health status, body-mass-index-for-age z scores at 7 years and 11 years | | | | | | | | | | | | | | | | | | |

| **Supplementary Table S6** Comparisons of characteristics between children with and without classified timing of puberty among respondents by traits | | | | | | | | | | | | | | |
| --- | --- | --- | --- | --- | --- | --- | --- | --- | --- | --- | --- | --- | --- | --- |
|  | Growth spurt | | | |  | | Body hair growth | | |  | Skin changes | | | |
|  | Without (n=5450) | With (n=6433) | P value |  | | Without (n=8581) | | With (n=4302) | P value |  | Without (n=7492) | With (n=5311) | P value |  |
| **Child’s age at questionnaire completion** |  |  |  |  | |  | |  |  |  |  |  |  |  |
| at 11 years | 11.2±0.3 | 11.1±0.3 | <0.001 |  | | 11.2±0.3 | | 11.2±0.3 | <0.001 |  | 11.2±0.3 | 11.2±0.3 | 0.21 |  |
| at 14 years | 14.3±0.3 | 14.2±0.3 | <0.001 |  | | 14.3±0.3 | | 14.3±0.3 | 0.55 |  | 14.3±0.3 | 14.2±0.3 | 0.003 |  |
|  |  |  |  |  | |  | |  |  |  |  |  |  |  |
| **Maternal characteristics** |  |  |  |  | |  | |  |  |  |  |  |  |  |
| Education (n, %) |  |  | 0.007 |  | |  | |  | 0.38 |  |  |  | <0.001 |  |
| None/other | 1118 (20.6%) | 1163 (18.1%) |  |  | | 1672 (19.5%) | | 814 (19.0%) |  |  | 1510 (20.2%) | 970 (18.3%) |  |  |
| GCSE D-G | 501 (9.2%) | 659 (10.3%) |  |  | | 855 (10.0%) | | 425 (9.9%) |  |  | 746 (10.0%) | 529 (10.0%) |  |  |
| GCSE A-C | 1801 (33.1%) | 2135 (33.3%) |  |  | | 2816 (32.9%) | | 1452 (33.8%) |  |  | 2530 (33.9%) | 1712 (32.3%) |  |  |
| A-level/diploma | 1028 (18.9%) | 1230 (19.2%) |  |  | | 1669 (19.5%) | | 788 (18.4%) |  |  | 1412 (18.9%) | 1021 (19.3%) |  |  |
| First/higher degree | 987 (18.2%) | 1230 (19.2%) |  |  | | 1549 (18.1%) | | 811 (18.9%) |  |  | 1273 (17.0%) | 1066 (20.1%) |  |  |
| OECD equivalized family income (n, %) |  |  | 0.002 |  | |  | |  | 0.32 |  |  |  | 0.038 |  |
| Q1 | 909 (16.7%) | 936 (14.6%) |  |  | | 1367 (15.9%) | | 640 (14.9%) |  |  | 1208 (16.1%) | 795 (15.0%) |  |  |
| Q2 | 1022 (18.8%) | 1133 (17.6%) |  |  | | 1557 (18.2%) | | 812 (18.9%) |  |  | 1400 (18.7%) | 951 (17.9%) |  |  |
| Q3 | 1091 (20.1%) | 1308 (20.3%) |  |  | | 1764 (20.6%) | | 852 (19.8%) |  |  | 1534 (20.5%) | 1068 (20.1%) |  |  |
| Q4 | 1133 (20.8%) | 1462 (22.7%) |  |  | | 1842 (21.5%) | | 960 (22.3%) |  |  | 1569 (21.0%) | 1223 (23.1%) |  |  |
| Q5 | 1286 (23.6%) | 1590 (24.7%) |  |  | | 2044 (23.8%) | | 1032 (24.0%) |  |  | 1773 (23.7%) | 1268 (23.9%) |  |  |

| **Supplementary Table 6** Comparisons of characteristics between children with and without classified timing of puberty among respondents by traits (continued) | | | | | | | | | | | | | | |
| --- | --- | --- | --- | --- | --- | --- | --- | --- | --- | --- | --- | --- | --- | --- |
|  | Voice breaking | | | |  | | Facial hair growth | | |  | Breast development | | | |
|  | Without (n=4492) | With (n=2105) | P value |  | | Without (n=4597) | | With (n=2000) | P value |  | Without (n=3014) | With (n=3163) | P value |  |
| **Child’s age at questionnaire completion** |  |  |  |  | |  | |  |  |  |  |  |  |  |
| at 11 years | 11.2±0.3 | 11.1±0.3 | <0.001 |  | | 11.1±0.3 | | 11.2±0.3 | <0.001 |  | 11.2±0.3 | 11.1±0.3 | 0.004 |  |
| at 14 years | 14.3±0.3 | 14.2±0.3 | <0.001 |  | | 14.2±0.3 | | 14.3±0.3 | <0.001 |  | 14.3±0.3 | 14.2±0.3 | <0.001 |  |
|  |  |  |  |  | |  | |  |  |  |  |  |  |  |
| **Maternal characteristics** |  |  |  |  | |  | |  |  |  |  |  |  |  |
| Education (n, %) |  |  | 0.007 |  | |  | |  | 0.15 |  |  |  | 0.051 |  |
| None/other | 875 (19.5%) | 370 (17.7%) |  |  | | 846 (18.5%) | | 399 (20.0%) |  |  | 648 (21.5%) | 591 (18.7%) |  |  |
| GCSE D-G | 466 (10.4%) | 203 (9.7%) |  |  | | 481 (10.5%) | | 185 (9.3%) |  |  | 290 (9.6%) | 314 (9.9%) |  |  |
| GCSE A-C | 1521 (33.9%) | 692 (33.0%) |  |  | | 1569 (34.3%) | | 646 (32.3%) |  |  | 942 (31.3%) | 1061 (33.6%) |  |  |
| A-level/diploma | 860 (19.2%) | 399 (19.0%) |  |  | | 873 (19.1%) | | 386 (19.3%) |  |  | 575 (19.1%) | 586 (18.6%) |  |  |
| First/higher degree | 760 (17.0%) | 431 (20.6%) |  |  | | 810 (17.7%) | | 381 (19.1%) |  |  | 553 (18.4%) | 604 (19.1%) |  |  |
| OECD equivalized family income (n, %) |  |  | 0.030 |  | |  | |  | 0.74 |  |  |  | 0.13 |  |
| Q1 | 709 (15.8%) | 294 (14.0%) |  |  | | 718 (15.6%) | | 287 (14.4%) |  |  | 505 (16.8%) | 488 (15.5%) |  |  |
| Q2 | 834 (18.6%) | 375 (17.8%) |  |  | | 839 (18.3%) | | 367 (18.4%) |  |  | 574 (19.0%) | 577 (18.3%) |  |  |
| Q3 | 951 (21.2%) | 413 (19.6%) |  |  | | 951 (20.7%) | | 412 (20.7%) |  |  | 609 (20.2%) | 598 (18.9%) |  |  |
| Q4 | 961 (21.4%) | 482 (22.9%) |  |  | | 1002 (21.8%) | | 440 (22.1%) |  |  | 623 (20.7%) | 705 (22.3%) |  |  |
| Q5 | 1032 (23.0%) | 538 (25.6%) |  |  | | 1084 (23.6%) | | 489 (24.5%) |  |  | 703 (23.3%) | 789 (25.0%) |  |  |
